# Effects of Dietary Supplementation with *Wolffia globosa* and *Limosilactobacillus reuteri* KUB-AC5 on Health Parameters and Gut Microbiota Composition in Dogs

**DOI:** 10.3390/biology15131067

**Published:** 2026-07-03

**Authors:** Sathita Areerat, Attawit Kovitvadhi, Koramit Jenjirawatn, Surangkhalak Khamma, Peeraya Chapanon, Pipatpong Chundang, Napat Praditrungwatana, Nichaphon Pliantiangtam, Preecha Patumcharoenpol, Nattaphong Akrimajirachoote, Massalin Nakphaichit, Suvimol Charoensiddhi, Tuchakorn Lertwanakarn, Pornsucha Palaseweenun

**Affiliations:** 1 Department of Physiology, Faculty of Veterinary Medicine, Kasetsart University, Bangkok 10900, Thailand; sathita.a@ku.th (S.A.); pichandang@gmail.com (P.C.); nattaphong.a@ku.th (N.A.); tuchakorn.l@ku.th (T.L.); 2 Independent Researcher, Lam Luk Ka 12130, Thailand; attawitthai@gmail.com; 3 Graduate Student in Animal Health and Biomedical Science Program, Faculty of Veterinary Medicine, Kasetsart University, Bangkok 10900, Thailand; koramit.jen@mahidol.ac.th (K.J.); surangkhalak@gmail.com (S.K.); peeraya.chap@ku.th (P.C.); minkupraditst22@gmail.com (N.P.); nichaphon.p@ku.th (N.P.); 4 Department of Pre-Clinic and Applied Animal Science, Faculty of Veterinary Science, Mahidol University, Nakhon Pathom 73170, Thailand; 5 Medical Bioinformatics, Siriraj Genomics, Faculty of Medicine Siriraj Hospital, Mahidol University, Bangkok 10700, Thailand; preecha.pat@mahidol.ac.th; 6 Department of Biotechnology, Faculty of Agro-Industry, Kasetsart University, Bangkok 10900, Thailand; fagimln@ku.ac.th; 7 Department of Food Science and Technology, Faculty of Agro-Industry, Kasetsart University, Bangkok 10900, Thailand; suvimol.ch@ku.th

**Keywords:** *Wolffia globosa*, *Limosilactobacillus reuteri*, gut microbiota, probiotic, prebiotic, synbiotic, dogs

## Abstract

The aquatic plant *Wolffia globosa* and the probiotic *Limosilactobacillus reuteri* KUB-AC5 have been proposed as candidate synbiotic components targeting gut microbiota modulation. In this study, we investigated the effects of *W. globosa* supplementation, alone or in combination with *L. reuteri* KUB-AC5, on the gut microbiota and health status in healthy adult dogs. For a 28-day feeding trial, 24 healthy dogs were randomly assigned to three dietary treatment groups: control, prebiotic (PRE; 3.5% *W. globosa* powder), and synbiotic (SYN, 3.5% *W. globosa* powder combined with *L. reuteri* KUB-AC5 at 1 × 10^8^ CFU/day). No significant differences were observed in body weight, body condition score, fecal consistency, feed intake, or blood parameters among groups (*p* > 0.05). Although overall gut microbial diversity remained unchanged, linear discriminant analysis effect size analysis revealed the selective enrichment of specific bacterial taxa. The PRE group exhibited higher relative abundances of *Parabacteroides merdae*, *Romboutsia lituseburensis*, *Subdoligranulum variabile*, and *Phocaeicola sartorii*, whereas the SYN group showed increased levels of *Escherichia coli* and *Terrisporobacter glycolicus* and reduced levels of *Anaerobiospirillum succiniciproducens* and *Campylobacter upsaliensis* (*p* < 0.05). In conclusion, *W. globosa*, alone or combined with *L. reuteri* KUB-AC5, was safely used to modulate gut bacterial composition as a potential functional ingredient.

## Simple Summary

Functional dietary ingredients that support gut health are of growing interest in companion animal research. Watermeal or duckweed (*Wolffia globosa*) is a nutrient-rich aquatic plant with potential prebiotic properties, while *Limosilactobacillus reuteri* KUB-AC5 is a beneficial probiotic bacterium. In this study, we investigated the effects of *W. globosa* alone and in combination with *L. reuteri* KUB-AC5 on gut microbiota composition and health status in healthy adult dogs during a 28-day feeding trial. No significant changes were observed in body weight, body condition score, feed intake, fecal consistency, or hematological and biochemical parameters, indicating that both treatments were well tolerated. Overall gut microbial diversity did not differ significantly among groups; however, specific bacterial taxa differed between dogs depending on whether they received the prebiotic or synbiotic diet. These findings suggest that *W. globosa*, either alone or combined with *L. reuteri* KUB-AC5, is safe for adult dogs and may exert prebiotic and synbiotic effects by selectively modulating gut microbial composition.

## 1. Introduction

The gut microbiota has emerged as a key determinant of gastrointestinal and systemic health in animals. This complex microbial ecosystem, composed of bacteria, viruses, archaea, protozoa, and fungi, colonizes the gastrointestinal tract and performs essential physiological functions. Among these microorganisms, bacteria represent the dominant and most extensively studied group [1]. Under balanced conditions, the gut bacterial community contributes to digestion, nutrient metabolism, immune regulation, and resistance to colonization by pathogens [1,2,3]. Disruption of this microbial equilibrium, commonly referred to as dysbiosis, has been associated with numerous disease conditions in companion animals. In dogs, dysbiosis is strongly linked to chronic enteropathy and is characterized by increased dysbiosis index scores and altered microbial composition [4]. Moreover, accumulating evidence suggests that alterations in the gut microbiota may contribute to extraintestinal disorders, including chronic kidney disease and atopic dermatitis [5,6]. Therefore, maintaining microbial homeostasis is increasingly recognized as an important target for improving gastrointestinal health and overall physiological stability in dogs.

Dietary strategies targeting gut microbial modulation have attracted considerable attention in recent years. Among these approaches, prebiotics are one of the most widely studied functional dietary components. Prebiotics are non-digestible substrates selectively utilized by beneficial gut microorganisms, thereby improving microbial balance and host health [7]. Common prebiotics, including fructooligosaccharides (FOSs), galactooligosaccharides (GOSs), mannan-oligosaccharides (MOSs), and inulin, have been reported to enhance gastrointestinal function, stabilize gut microbiota, and support immune responses in animals [8,9]. As interest in microbiota-targeted nutrition continues to grow, the search for novel prebiotic ingredients with improved functionality and sustainability has become an active area of research.

Recently, increasing interest has focused on sustainable plant-based ingredients with potential prebiotic properties. *Wolffia globosa* (watermeal or duckweed), the smallest flowering aquatic plant, has attracted attention due to its high nutritional value, rapid biomass production [10,11], and sustainability-related advantages. *W. globosa* possesses a favorable nutritional composition rich in protein and dietary fiber, with protein content reported to exceed 45% on dry matter basis [10,12]. Authors of previous studies have demonstrated the nutritional and functional benefits of *W. globosa* when incorporated into human foods and livestock feeds [13,14,15]. Despite its traditional consumption in Southeast Asia and promising nutritional properties, information regarding the safety, physiological tolerance, and potential effects of *W. globosa* regarding gut microbial composition in dogs remains limited.

In addition to prebiotics, probiotics have been widely explored as dietary strategies for modulating gut microbiota. When combined with prebiotics, these formulations are referred to as synbiotics, and they may enhance probiotic survival, microbial activity, and host immune responses within the gastrointestinal tract [16]. *Limosilactobacillus reuteri* KUB-AC5, originally isolated from chicken intestines, produces bacteriocin-like antimicrobial peptides capable of inhibiting pathogenic bacteria while exerting minimal inhibitory effects on beneficial lactic acid bacteria [17,18]. Previous in vitro studies have demonstrated that *W. globosa* can support the growth and viability of *L. reuteri* KUB-AC5 while enhancing organic acid production and suppressing *Salmonella* spp. under experimental conditions [19]. However, the effects of this synbiotic combination on gut microbiota composition and host health in dogs have not been investigated. In this study, we investigated the effects of *W. globosa* supplementation, alone or in combination with *L. reuteri* KUB-AC5, on gut microbiota composition and health status in dogs. Specifically, the hematological parameters, safety outcomes, and alterations in gut bacterial composition were evaluated to assess the potential of this novel prebiotic and synbiotic approach for improving canine gut health.

## 2. Materials and Methods

### 2.1. Animals and Husbandry

This study was conducted at a private dog farm in Nakhon Nayok Province, Thailand. A total of twenty-four healthy neutered adult Thai mixed-breed dogs (10 males and 14 females), aged between 2 and 7 years, were enrolled in this study (Table S1). The dogs had an initial body weight (BW) of 16.58 ± 3.23 kg and a body condition score (BCS; 9-point scale) of 4.08 ± 1.31 (mean ± standard error of the mean; SEM). Before the experiment, all dogs underwent a complete physical examination performed by a licensed veterinarian, and no clinical abnormalities were detected. Throughout this study, dogs were individually housed in open-system pens measuring 2 × 2 × 3 m^3^, and the average ambient temperature and relative humidity during the study period were approximately 33–35 °C and 55–75%, respectively. All animals were fed a commercial kibble diet as a basal diet (Smillie^®^, Petpal Products Co., Ltd., Saraburi, Thailand), containing 20% crude protein, 8.9% crude fat, 6.7% crude fiber, and metabolizable energy of 3360 kcal/kg dry matter basis (DM). The experimental procedures were approved by the Institutional Animal Care and Use Committee of Kasetsart University (Protocol No. ACKU68-VET-016).

### 2.2. Experimental Design

A total of 80 dogs were screened, and 24 eligible dogs were enrolled in the study. The dogs were randomly assigned to one of three dietary treatment groups (*n* = 8 per group): control (CON), prebiotic (PRE), and synbiotic (SYN), using a simple randomization procedure, and were subsequently housed according to a completely randomized design (CRD). Dogs in the CON group were supplemented with maltodextrin as a placebo. The PRE group was supplemented with 3.5% *W. globosa* powder (Flo Wolffia^®^, Advanced Greenfarm Co., Ltd., Nakhon Pathom, Thailand), corresponding to approximately 650 mg/kg BW/day; this inclusion level was selected to avoid the reduced palatability reported at higher duckweed inclusion rates. The nutrient values reported for the *W. globosa* powder were measured from the Food and Nutrition Laboratory Institute of Nutrition, Mahidol University, using standard AOAC International (2019) methods [20] containing 40.38% crude protein (992.23), 6.18% total fat (992.06), 38.42% total carbohydrates (including sugars and dietary fiber), 26.88% total dietary fiber (985.29), 16.71% insoluble dietary fiber (991.42), 10.17% soluble dietary fiber (993.19), and 18.28% ash (930.30) on dry weight. The SYN group received the same level of *W. globosa* supplementation combined with *L. reuteri* KUB-AC5 (1 g; 1 × 10^8^ CFU/day), originally isolated from the chicken intestine [21,22], and prepared as previously described by Tantibhadrasapa et al. [23]. This dosage was selected based on previous reports demonstrating that Lactobacillus populations in canine feces generally range from 10^6^ to 10^8^ CFU/g feces [24], thereby representing a physiologically relevant supplementation level for dogs. All supplements were administered daily as top dressing and mixed thoroughly with the kibble immediately before feeding.

The feeding trial was conducted for 28 days. Dogs were fed once daily at 15:00 h, and the daily food allowance was calculated based on the estimated energy requirement (1.6 × 70 × BW^0.75^) [25]. Fresh water was provided *ad libitum* throughout the experimental period. Leftover feed was collected and weighed at 09:00 h the following day to determine daily feed intake. Body weight and BCS [26] were evaluated on Days 0, 14, and 28.

### 2.3. Sample Collection

Blood samples (3 mL) were obtained via the venipuncture of the cephalic vein in the forelimb of each dog on Day 0 (baseline) and Day 28 to compare the effects of supplementation on hematological and serum biochemical profiles. All blood samples were analyzed at the Veterinary Diagnostic Laboratory of Kasetsart University Veterinary Teaching Hospital (Bangkok, Thailand). Hematological parameters were analyzed using an automated hematology analyzer (Sysmex XN-1000, Sysmex Corporation, Kobe, Japan) to determine complete blood count (CBC) parameters, including white blood cells (WBCs), neutrophils (NEU), lymphocytes (LYM), monocytes (MONO), eosinophils (EO), basophils (BASO), red blood cells (RBCs), hemoglobin (HGB), hematocrit (HCT), mean corpuscular volume (MCV), mean corpuscular hemoglobin (MCH), mean corpuscular hemoglobin concentration (MCHC), the percentage of red cell distribution width (RDW%), platelets (PLT), and mean platelet volume (MPV). Following collection, blood samples were allowed to clot for 30 min and centrifuged at 3000× *g* for 10 min to obtain serum. Serum biochemical parameters were measured using an automated clinical chemistry analyzer (ILab TAURUS, Instrumentation Laboratory, Milan, Italy) with commercial reagent kits supplied by the manufacturer, including blood urea nitrogen (BUN), creatinine (CREA), total protein (TP), albumin (ALB), globulin (GLOB), alanine aminotransferase (ALT), and alkaline phosphatase (ALP).

Fecal samples were collected daily at 10:00 h for fecal consistency scoring and microbial analysis. Fecal score (FS) was assessed using the Purina Fecal Scoring System (Nestlé Purina PetCare, St. Louis, MO, USA), with scores ranging from 1 (very hard/dry) to 7 (watery with no texture). Clinical assessments were performed by the same trained evaluator throughout the study. Assessments were not conducted in a blinded manner, and photographic records were not collected. Samples designated for microbial analysis were immediately frozen at −20 °C and stored until further processing.

### 2.4. DNA Extraction and Sequencing

DNA was extracted using the MGIEasy Magnetic Beads Genomic DNA Extraction Kit (MGI Tech Co., Ltd., Shenzhen, China) according to the manufacturer’s protocol. The 16S rRNA genes were amplified using the primer pair 16S-F, GTCTCGTGGGCTCGG + AGAGTTTGATCCTGGCTCAG, and 16S-R, TCGTCGGCAGCGTC + GGTTACCTTGTTACGACTTC. Sequencing was performed using the Oxford Nanopore Technologies (ONT; Oxford, UK) PromethION platform using R10.4.1 flow cells, and at least 50,000 reads were obtained from each sample. Basecalling and demultiplexing were performed using ONT’s Dorado (https://github.com/nanoporetech/dorado, accessed on 12 January 2026). The primer and adapter sequence was trimmed using pychopper (version 2, https://github.com/epi2me-labs/pychopper, accessed on 15 January 2026), and the reads were then filtered using chopper [27]. Reads with a median quality score < Q10 or a length outside the range of 1200–1800 base pairs were discarded. The clean reads were taxonomically classified to the species level using emu [28]. A custom Python (version 3.12) script was then used to translate the emu output files into abundance tables for each taxonomic level (e.g., family, genus, and species).

### 2.5. Statistical Analysis

All data analyses and visualizations were performed using R statistics within RStudio IDE (version 2025.05.1+513). Statistical significance was set at *p* < 0.05. The characteristics of each dog and their feed intake are described using descriptive statistics. Normality and homogeneity of variance were assessed using the Shapiro–Wilk test and Levene’s test, respectively. BW, BCS, FS, hematological parameters, and serum biochemical parameters were analyzed using two-way mixed ANOVA, with group set as the between-subject factor and collection time set as the within-subject factor. The Bonferroni test and Duncan’s multiple range test were used to perform post hoc analyses of the within- and between-subject variables, respectively, utilizing Rcmdr (version 2.9-5) and ggplot2 (version 3.5.2).

Changes in the detected bacterial species were used to calculate the beta and alpha diversity, with the groups and collection dates used as factors. Beta diversity was assessed using the Bray–Curtis dissimilarity index calculated from the relative abundance at the species level. A principal coordinates analysis (PCoA) was performed on the Bray–Curtis dissimilarity matrix to visualize differences in community composition between groups and over time (ggplot2 version 3.5.2 and ggforce version 0.5.0). The percentages of variance explained by the first two PCoA axes are reported. Differences in bacterial communities across groups and collection times were statistically evaluated using a permutational multivariate analysis of variance (PERMANOVA) with 999 permutations (adonis2 function with vegan package version 2.5-6). Alpha diversity indices, including the Chao1, Shannon–Weaver, and Simpson’s indices, were calculated using the vegan package (version 2.5-6). The Firmicutes/Bacteroidetes Ratio (F/B ratio) and the relative abundances of bacterial taxa at the phylum and species levels and of all *Lactobacillus* sp. were analyzed using two-way mixed model ANOVA with group as the between-subject factor and collection time as the within-subject factor with centered log-ratio transformation. Post hoc comparisons were conducted using Duncan’s multiple range test (Rcmdr package version 2.9-5). Heatmaps were generated using the complete dataset at the phylum level and the top 50 taxa at the species level. All data were transformed using the centered log-ratio transformation before performing analysis using the pheatmap (version 1.0.13), compositions (version 2.0-9), and RColorBrewer (version 1.1-3) packages. Taxa that differed in abundance between groups on Day 28 were identified at the species level using linear discriminant analysis effect size (LEfSE) analysis (lefser; version 1.18.0).

## 3. Results

### 3.1. Feed Intake, BW, BCS, and Fecal Score

Two dogs, one from the PRE group and one from the SYN group, were withdrawn from this study on Day 14 due to progressively reduced feed intake during the first two weeks of supplementation. By Day 14, their energy intake had declined to approximately 54.7% and 54.3% of their calculated daily energy requirements (DER), respectively. Consequently, data from the remaining 22 dogs were included in the final analyses. Data obtained from the remaining dogs are summarized in Table 1. No significant effects of day, dietary treatment, or their interaction were observed for BW, BCS, FS, or feed intake throughout the 28-day study period (*p* > 0.05). Body weight and BCS remained stable within the ideal range (BCS 4–5/9), while FS were consistently maintained within the normal range (2–3/7). Feed intake was also comparable among all experimental groups.

### 3.2. Hematology and Serum Biochemistry Profiles

All hematological and serum biochemical parameters remained within the established reference ranges across groups (Table 2). Time effects were observed, with NEU, BASO, erythrocyte indices (RBC, HGB, HCT, MCV, and RDW), BUN, CREA, and ALP increasing after 28 days, while MCHC, TP, GLOB, and ALT decreased after 28 days. No effects of group and day × group interaction were detected.

### 3.3. Analysis of Fecal Bacteria

**Figure 1:**
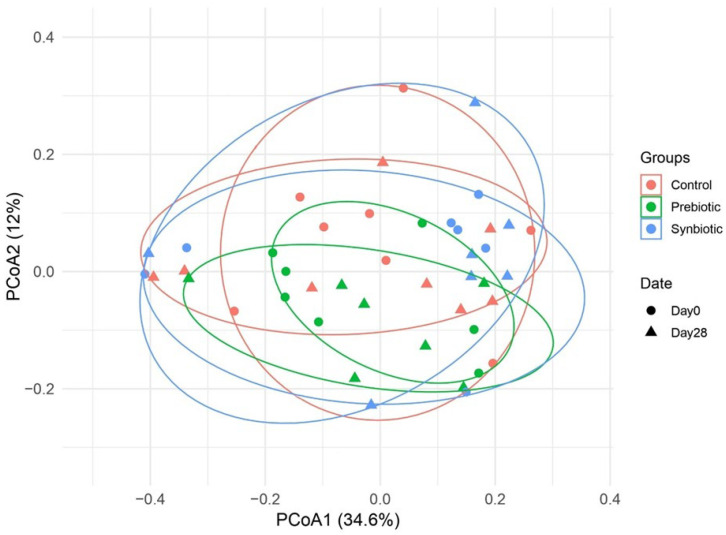
Principal coordinate analysis (PCoA) plot illustrating the beta diversity of the gut bacterial communities in dogs from the CON, PRE, and SYN groups before (Day 0) and after 28 days of dietary intervention.

The beta diversity of the gut bacterial communities, as shown by PCoA (Figure 1), did not show any distinct clusters among groups or time points. PERMANOVA revealed no statistical difference (*p* > 0.05).

The results for the alpha diversity indices, including the Chao1 richness and Shannon–Weaver and Simpson’s indices, are presented in Table 3. No significant changes were observed between Days 0 and 28 across the CON, PRE, and SYN groups.

**Figure 2:**
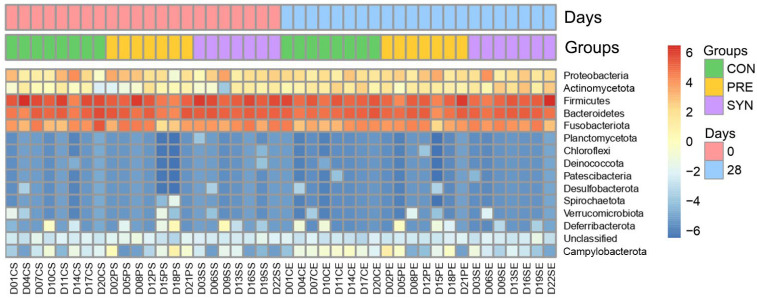
Heatmap of the relative abundances of the bacterial phyla in dogs from the CON, PRE, and SYN groups before (Day 0) and after 28 days of dietary intervention.

**Figure 3:**
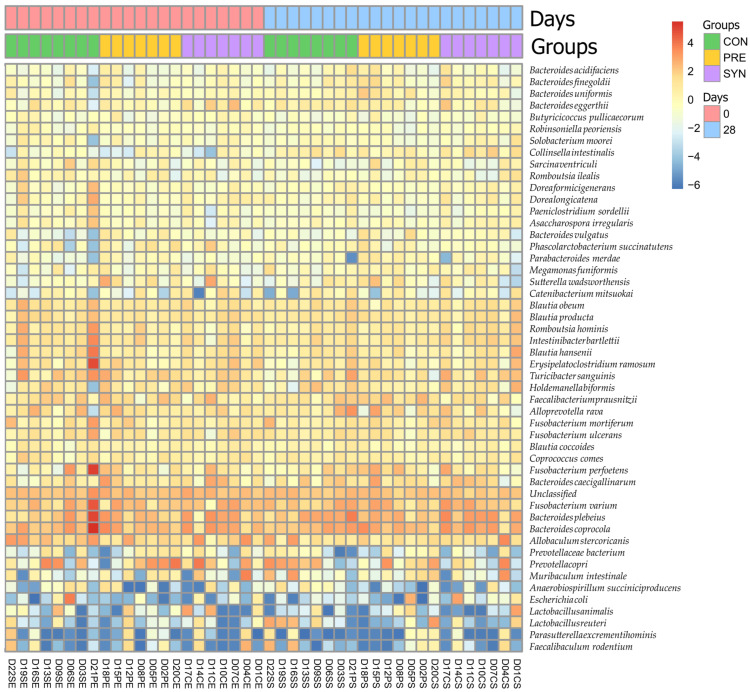
Heatmap of the relative abundances of the bacterial species in dogs from the CON, PRE, and SYN groups before (Day 0) and after 28 days of dietary intervention.

Figure 2 and Figure 3 present the heatmaps of microbial composition at the phylum and species levels, respectively. At the phylum level (Figure 2), Firmicutes, Bacteroidetes, and Fusobacteriota were the predominant taxa across all experimental groups. At the species level (Figure 3), distinct variations in microbial composition were observed among dietary groups and sampling time points.

The relative abundance of bacterial phyla and species was compared across groups, with the complete dataset presented in Appendix A. At the phylum level, most taxa remained unchanged throughout the 28-day intervention (*p* > 0.05), except for Actinomycota, which increased significantly over time (*p* < 0.001). At the species level, the abundance of *Phocaeicola plebeius* increased significantly over the study period (*p* < 0.05), whereas that of *Erysipelatoclostridium ramosum* significantly decreased (*p* < 0.05). A significant interaction between the study factors was observed for *Romboutsia hominis* (*p* < 0.05); its abundance was similar among groups at Day 0 but differed at Day 28, with higher values recorded in the SYN group.

### 3.4. Linear Discriminant Analysis Effect Size (LEfSE) Analysis

**Figure 4:**
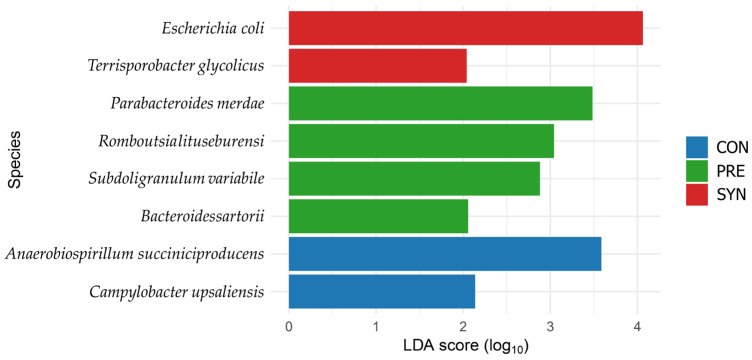
Linear discriminant analysis effect size (LEfSE) of bacterial species that were differentially abundant in the gut microbiota of dogs in the CON, PRE, and SYN groups on Day 28. Species with a linear discriminant analysis (LDA) score (log_10_) > 2.0 were considered significantly enriched (*p* < 0.05).

LEfSe analysis identified bacterial biomarkers differentiating the three treatment groups (LDA score > 2.0; Figure 4). In the SYN group, *Escherichia coli* and *Terrisporobacter glycolicus* were enriched, with *E. coli* exhibiting the highest LDA score (~4.0). The PRE group showed higher abundances of *Parabacteroides merdae*, *R. lituseburensi*, *Subdoligranulum variabile*, and *P. sartorii*. Conversely, in the CON group, *Anaerobiospirillum succiniciproducens* and *Campylobacter upsaliensis* were enriched.

## 4. Discussion

The strategic application of probiotics, prebiotics, and synbiotics in animal nutrition has emerged as an effective approach for positively modulating the gastrointestinal microenvironment. In recent years, the pet food industry has undergone a paradigm shift toward functional nutrition, largely driven by increasing pet owner awareness regarding companion animal health and well-being. As previously described, both prebiotics and probiotics play important roles in promoting host health. Furthermore, the incorporation of functional ingredients possessing prebiotic properties has demonstrated synergistic effects in supporting health and maintaining microbial homeostasis in both animals and humans [29,30,31].

In this study, supplementation with *W. globosa*, either alone or in combination with *L. reuteri* KUB-AC5, was well tolerated in healthy dogs, with no significant changes in clinical or biochemical parameters, supporting previous evidence regarding the safety of prebiotic and symbiotic interventions [32,33]. However, the rejection of the *W. globosa*-supplemented diet by two dogs indicates a potential challenge related to the palatability and individual acceptance of duckweed as a novel dietary ingredient. This observation is consistent with previous findings in other duckweed (*Lemna* sp.) species [34], where reduced palatability was associated with lower intake and preference, potentially due to the presence of secondary metabolites such as oxalic acid and phenolic compounds that contribute to off-odors and bitter taste. Therefore, the incorporation of *W. globosa* into dog diets may require the use of processing strategies to reduce undesirable compounds, the use of palatability enhancers, or the extraction of purified compounds to improve dietary acceptance.

Overall, dietary supplementation did not induce marked alterations in gut microbiota diversity. Both PRE and SYN treatments demonstrated selective modulation of specific bacterial taxa without substantially affecting the overall microbial community structure. Moreover, in the PRE group, the enrichment of *P. merdae*, *R. lituseburensis*, *S. variabile*, and *P. sartorii* suggested the occurrence of targeted microbial modulation associated with *W. globosa* supplementation. Notably, *P. merdae* has been associated with branched-chain amino acid (BCAA) metabolism, and its increased abundance may reflect adaptation to the relatively high BCAA content of *W. globosa*. This observation may be physiologically relevant, as elevated BCAA metabolism has been linked to metabolic disorders, including insulin resistance and obesity [35,36]. Additionally, *P. merdae* has been reported to exert cardioprotective effects in humans [35]. Other enriched taxa, including *R. lituseburensis* and *S. variabile*, have been associated with immune modulation and improved metabolic profiles [37,38,39], whereas *P. sartorii* has been linked to anti-obesity effects in murine models [40]. Although the functional significance of these taxa in dogs remains unclear, the observed microbial shifts suggest the existence of potential metabolic and immunomodulatory implications that warrant further investigation.

Despite these selective microbial changes, no broad prebiotic effects of *W. globosa* were observed in this study. This likely reflects the limited fermentability of *W. globosa* in its biomass form. This is consistent with the findings of Pinna et al. [41], who reported that fecal microbiota composition in dogs remained unaltered following the inclusion of intact seaweed at a proportion of 1.5% of the diet. The structural complexity of the biomass may restrict microbial accessibility and fermentation, limiting its capacity to induce substantial microbiome shifts compared with purified or highly fermentable prebiotic substrates reported in previous studies [9]. In addition, *W. globosa* contains only 26.88% of total dietary fiber, which is considered a prebiotic substrate, and this is insufficient to drive pronounced microbiome modulation. However, the interpretation of these findings should consider the relatively small sample size and inherent inter-individual variability in canine gut microbiota. Variation in breed and age among dogs may have further contributed to differences in microbiota composition and physiological response, despite standardized diet and housing conditions. Together, these findings suggest that the substrate matrix, dosage, sample size, and population heterogeneity represent key limiting factors in this study.

The biological relevance of observed taxa shifts required careful interpretation. Although *E. coli* was enriched in the SYN group, this common commensal in the canine gut contributes to carbohydrate metabolism, and few strains are potentially pathogenic [42]. Although *E. coli* was enriched in the SYN group, this finding should be interpreted cautiously. *E. coli* includes both commensal and pathogenic strains, and the 16S rRNA sequencing approach used in this study cannot distinguish between strain types. Therefore, the biological relevance of this increase remains unclear and does not necessarily indicate either a beneficial or detrimental effect on gut health. Similarly, the enrichment of *T. glycolicus* following supplementation is considered part of the indigenous canine microbiota, despite its role remaining incompletely characterized and context-dependent [43,44,45]. While some evidence suggests potential involvement in antimicrobial production [46], its functional significance in dogs remains poorly characterized.

These composition shifts likely reflect microbial responsiveness to symbiotic intervention but do not necessarily translate into functional or clinical outcomes, emphasizing the need for targeted investigation into strain–host interactions. Although LEfSe identified several differentially abundant taxa, no significant differences were observed in alpha diversity, beta diversity, or the abundance of major bacterial taxa. Therefore, these taxonomic differences should be interpreted cautiously and considered exploratory, as they do not necessarily indicate a broad or biologically robust shift in the overall gut microbiota [47]. The relatively small sample size may have limited our ability to detect subtle treatment effects, particularly given the high inter-individual variability in gut microbiota data. Consequently, the absence of significant differences should not necessarily be interpreted as evidence of a complete lack of biological response. Moreover, the 28-day intervention period may have been insufficient to capture longer-term functional microbiome adaptations or stable metabolic outcomes. Studies with intervention periods of at least 6 months [48] are warranted to determine whether the observed microbial changes persist and translate into meaningful physiological effects. In addition, baseline microbiome profiling was not performed before intervention, limiting formal verification of group equivalence and potentially contributing to variability in treatment response. Incorporating baseline microbiome profiling in future studies would strengthen causal interpretation of treatment effects.

In parallel, probiotic persistence is a critical factor influencing synbiotic efficacy. Although supplemented *L. reuteri* KUB-AC5 probiotics are expected to persist within the gastrointestinal tract to exert their beneficial functions in suppressing members of the Enterobacteriaceae family [20,49,50], no detectable increase in *L. reuteri* levels was observed in our study. This finding suggests limited persistence and colonization of probiotics strains in the canine gastrointestinal tract. A likely explanation is host-specificity, as *L. reuteri* KUB-AC5 was originally isolated from chicken intestines [18]. Differences in gut environment, including pH, resident microbiota composition, and strain-specific tolerances, may have further hindered the survival and establishment of *L. reuteri* KUB-AC5 in the canine gut [51,52]. Consequently, the microbial responses observed in the SYN group may have been driven primarily by the *W. globosa* component. However, transient or localized effects of *L. reuteri* KUB-AC5 that were not captured by fecal microbiota analysis cannot be excluded. In addition, species-level 16S rRNA sequencing cannot distinguish the administered *L. reuteri* KUB-AC5 strain from resident *L. reuteri* populations. Therefore, the absence of strain-specific tracking limited the ability to confirm colonization or persistence of *L. reuteri* KUB-AC5 in the canine gastrointestinal tract, thereby restricting functional interpretation of probiotic effects.

## 5. Conclusions

The findings of this study demonstrate that *W. globosa*, either alone or in combination with *L. reuteri* KUB-AC5, was safe and well-tolerated in healthy dogs over the 28-day study period. However, the supplementation had only limited effects on the overall gut microbiota, as no significant changes were observed in alpha diversity, beta diversity, or the abundance of most bacterial taxa. Furthermore, stable colonization of the supplemented *L. reuteri* KUB-AC5 strain was not demonstrated. The rejection of the *W. globosa*-supplemented diet by two dogs also suggests that palatability and individual acceptance may be important considerations when incorporating duckweed as a novel dietary ingredient. Future studies incorporating larger sample sizes, longer intervention periods, baseline microbiome profiling, and strain-specific approaches are warranted to better characterize the long-term physiological and microbiological effects of *W. globosa* supplementation in dogs.

## Figures and Tables

**Table 1:** Effects of dietary supplementation on BW, BCS, FS, and feed intake in dogs (*n* = 22).

Parameters	Day			Group			SEM ^1^	*p*-Value		
	0	14	28	CON	PRE	SYN		Day	Group	Day × Group
BW (kg)	16.58	16.63	15.06	16.85	14.74	16.68	0.83	0.158	0.416	0.567
BCS	4.00	4.14	4.14	4.29	3.62	4.33	0.28	0.505	0.496	0.600
FS ^2^	2.58	2.53	2.47	2.67	2.53	2.33	0.14	0.642	0.301	0.264
Feed intake (% DER ^3^)										
Period 1–14	-	-	-	93.8	84.8	90.4	3.040	-	0.498	-
15–28	-	-	-	99.8	89.2	90.1	2.924	-	0.262	-
1–28	-	-	-	96.8	87.0	90.2	2.725	-	0.334	-

^1^ Standard error of the mean. ^2^ Purina Fecal Scoring System (Nestlé Purina PetCare, St. Louis, MO, USA). ^3^ Daily energy requirement.

**Table 2:** Hematological and serum biochemical profiles of dogs in the CON, PRE, and SYN groups (*n* = 22).

Parameters ^1^	Day		Group			SEM ^2^	*p*-Value			Reference Range ^3^
	0	28	CON	PRE	SYN		Day	Group	Day × Group	
WBCs (10^3^/µL)	12.37	13.49	12.95	13.69	12.14	0.824	0.07	0.753	0.289	5.05–16.80
NEU (10^3^/µL)	7.50	8.70	8.07	8.65	7.60	0.560	0.028	0.743	0.595	2.95–11.60
LYM (10^3^/µL)	2.97	2.88	2.96	3.17	2.64	0.252	0.497	0.692	0.130	1.05–5.10
MONO (10^3^/µL)	0.44	0.49	0.46	0.50	0.43	0.033	0.07	0.701	0.440	0.16–1.12
EOS (10^3^/µL)	1.43	1.36	1.42	1.32	1.45	0.146	0.571	0.931	0.249	0.06–1.23
BASO (10^3^/µL)	0.03	0.04	0.03	0.04	0.03	0.005	0.002	0.490	0.025	0.00–0.10
RBCs (10^6^/µL)	7.07	7.49	7.37	7.25	7.20	0.181	0.009	0.921	0.555	5.65–8.87
HGB (g/dL)	16.02	17.08	16.70	16.31	16.62	0.360	0.007	0.881	0.536	13.1–20.5
HCT (%)	45.72	49.94	47.86	47.45	48.18	0.824	<0.001	0.927	0.475	37.3–61.7
MCV (fL)	65.07	66.96	65.34	65.76	67.05	0.729	<0.001	0.606	0.916	61.6–73.5
MCH (pg)	22.71	22.86	22.75	22.52	23.09	0.186	0.218	0.453	0.757	21.2–25.9
MCHC (g/dL)	34.96	34.17	34.86	34.32	34.48	0.228	0.003	0.562	0.941	32.0–37.9
RDW (%)	29.53	31.43	31.01	30.34	30.02	0.555	0.001	0.732	0.615	-
PLT (10^3^/µL)	227.14	225.50	212.88	239.36	228.64	15.017	0.929	0.625	0.726	148–484
MPV (fL)	11.14	11.41	11.22	11.36	11.26	0.147	0.081	0.911	0.868	-
BUN (mg/dL)	11.27	13.41	11.94	12.00	13.14	0.604	<0.001	0.644	0.809	10.0–26.0
CREA (mg/dL)	1.09	1.19	1.07	1.17	1.20	0.033	<0.001	0.210	0.770	0.50–1.30
TP (g/dL)	6.96	6.37	6.84	6.61	6.52	0.115	0.001	0.379	0.164	5.30–7.80
ALB (g/dL)	3.15	3.09	3.13	3.14	3.09	0.044	0.194	0.826	0.161	2.30–3.20
GLOB (g/dL)	3.80	3.28	3.71	3.46	3.44	0.106	<0.001	0.423	0.215	2.70–4.40
ALT (U/L)	42.14	31.55	26.56	37.00	48.43	4.697	0.016	0.144	0.360	6.00–70.0
ALP (U/L)	23.64	35.82	31.50	30.29	27.14	3.145	<0.001	0.847	0.606	20.0–108.0

^1^ WBCs, white blood cells; NEU, neutrophils; LYM, lymphocytes; MONO, monocytes; EOS, eosinophils; BASO, basophils; RBCs, red blood cells; HGB, hemoglobin; HCT, hematocrit; MCV, mean corpuscular volume; MCH, mean corpuscular hemoglobin; MCHC, mean corpuscular hemoglobin concentration; RDW, red cell distribution width; PLT, platelets; MPV, mean platelet volume; BUN, blood urea nitrogen; CREA, creatinine; TP, total protein; ALB, albumin; GLOB, globulin; ALT, alanine aminotransferase; ALP, alkaline phosphatase. ^2^ Standard error of the mean. ^3^ Reference values were derived from the Veterinary Diagnostic Laboratory of Kasetsart University Veterinary Teaching Hospital. *n* = 22 due to exclusion of two dogs (one PRE, one SYN group) withdrawn on Day 14 because of progressive feed refusal and insufficient dietary intake during supplementation.

**Table 3:** Alpha diversity indices of gut bacterial communities in the CON, PRE, and SYN groups.

Alpha Diversity Index	Day		Group			SEM ^1^	*p*-Value		
	0	28	CON	PRE	SYN		Day	Group	Day × Group
Chao1	292	296	298	308	275	13.78	0.839	0.561	0.414
Shannon–Weaver	5.30	5.38	5.31	5.48	5.23	0.072	0.419	0.207	0.305
Simpson’s	0.94	0.95	0.95	0.95	0.94	0.003	0.214	0.714	0.611

^1^ Standard error of the mean.

## Data Availability

The raw data supporting the conclusions of this article will be made available by the authors on request.

## Supplementary Materials

The following supporting information can be downloaded at: https://www.mdpi.com/article/10.3390/biology15131067/s1, Table S1: Animal Information and Signalment; Table S2: Sequencing depth statistics for all samples (Data Quality Control).

## Abbreviations

The following abbreviations are used in this manuscript:

°C	Degree(s) Celsius
16S rRNA	16S ribosomal Ribonucleic Acid
ALB	Albumin
ALP	Alkaline phosphatase
ALT	Alanine aminotransferase
AOAC	Association of Official Analytical Chemists
BASO	Basophils
BCAA	Branched-chain amino acid
BCS	Body condition score
BUN	Blood urea nitrogen
BW	Body weight
CBC	Complete blood count
CFU	Colony-Forming Unit
Co., Ltd.	Company Limited
CON	Control group
CREA	Creatinine
CRD	Completely randomized design
DM	Dry matter basis
DNA	Deoxyribonucleic acid
EO	Eosinophils
F/B ratio	Firmicutes/Bacteroidetes Ratio
FS	Fecal score
GLOB	Globulin
h	Hour
HCT	Hematocrit
HGB	Hemoglobin
Kg	Kilogram
LDA	Linear discriminant analysis
LEfSE	Linear discriminant analysis effect size
LYM	Lymphocytes
MCH	Mean corpuscular hemoglobin
MCHC	Mean corpuscular hemoglobin concentration
MCV	Mean corpuscular volume
mL	Milliliter
MONO	Monocytes
MPV	Mean platelet volume
*n*	Number
NEU	Neutrophils
*p*	p-value
PCoA	Principal coordinate analysis
PERMANOVA	Permutational multivariate analysis of variance
PLT	Platelets
PRE	Prebiotic group
RBCs	Red blood cells
RDW	Red cell distribution width
SEM	Standard error of the mean
SYN	Synbiotic group
TP	Total protein
WBCs	White blood cells

## Appendix A

**Table A1:** Relative abundances of bacterial phyla and species in the control (CON), prebiotic (PRE), and synbiotic (SYN) groups.

% Relative Abundance	Day		Group			SEM ^1^	*p*-Value		
	0	28	CON	PRE	SYN		Day	Group	Day × Group
**Phylum**									
Firmicutes	48.9	44.4	49.1	44.3	46.3	2.443	0.183	0.604	0.935
Bacteroidetes	35.0	37.9	34.7	38.6	36.4	1.610	0.242	0.399	0.246
Fusobacteriota	12.0	12.6	12.0	12.2	12.7	1.265	0.674	0.958	0.542
Proteobacteria	3.43	3.41	3.27	3.65	3.36	0.996	0.950	0.977	0.479
Actinomycetota	0.48	1.50	0.89	0.86	1.24	0.157	<0.001	0.450	0.191
Campylobacterota	0.11	0.12	0.08	0.25	0.02	0.052	0.998	0.087	0.548
Deferribacterota	0.09	0.05	0.04	0.13	0.03	0.036	0.427	0.290	0.773
F/B ratio ^2^	1.67	1.28	1.70	1.28	1.42	0.228	0.291	0.615	0.481
**Species**									
*Bacteroides coprocola*	7.91	8.20	7.64	8.15	8.44	0.926	0.704	0.923	0.432
*Phocaeicola plebeius*	6.09	8.35	7.31	5.96	8.38	0.907	0.010	0.518	0.080
*Prevotella copri*	7.35	4.93	6.21	7.71	4.49	1.636	0.253	0.643	0.650
*Fusobacterium varium*	5.45	5.50	5.32	5.13	5.99	0.648	0.953	0.759	0.966
*Allobaculum stercoricanis*	5.00	3.42	4.77	2.99	4.79	0.880	0.109	0.576	0.961
*Turicibacter sanguinis*	2.87	2.50	2.88	2.55	2.59	0.466	0.633	0.882	0.308
*Bacteroides caecigallinarum*	2.33	2.96	1.89	2.98	3.17	0.533	0.419	0.392	0.737
*Alloprevotella rava*	2.51	2.77	3.19	2.95	1.70	0.589	0.728	0.416	0.948
*Faecalibacterium prausnitzii*	2.47	2.72	2.35	3.70	1.77	0.431	0.501	0.114	0.457
*Fusobacterium perfoetens*	2.56	2.52	2.67	3.03	1.91	0.630	0.951	0.680	0.193
*Intestinibacter bartlettii*	2.20	2.02	2.22	1.72	2.39	0.268	0.632	0.456	0.200
*Blautia producta*	1.97	1.96	2.00	1.72	2.16	0.210	0.968	0.582	0.667
*Erysipelatoclostridium ramosum*	2.39	1.46	2.17	1.31	2.25	0.387	0.044	0.520	0.452
*Holdemanella biformis*	1.85	1.96	2.00	1.44	2.27	0.343	0.819	0.544	0.231
*Muribaculum intestinale*	1.65	2.01	1.40	1.34	2.82	0.841	0.647	0.705	0.115
*Blautia obeum*	1.86	1.80	2.11	1.54	1.79	0.201	0.885	0.217	0.670
*Blautia hansenii*	1.87	1.64	2.06	1.13	2.04	0.329	0.582	0.312	0.256
*Fusobacterium ulcerans*	1.78	1.63	1.58	1.56	1.99	0.204	0.636	0.376	0.863
*Romboutsia hominis*	1.64	1.66	1.73	1.35	1.86	0.248	0.840	0.578	0.024
*Fusobacterium mortiferum*	1.63	1.66	1.80	1.74	1.38	0.281	0.856	0.789	0.643
*Lactobacillus reuteri*	0.48	2.00	1.92	1.25	0.46	0.493	0.072	0.370	0.379
*Coprococcus comes*	1.23	1.12	1.26	1.15	1.10	0.127	0.539	0.821	0.966
*Escherichia coli*	0.98	1.34	1.19	0.93	1.36	0.816	0.679	0.957	0.252
*Bacteroides eggerthii*	1.19	1.06	1.08	0.72	1.58	0.216	0.610	0.120	0.213
*Sutterella wadsworthensis*	1.18	1.06	0.51	1.87	1.06	0.339	0.750	0.100	0.626
All *Lactobacillus* spp.	3.60	4.87	4.23	4.51	3.95	1.495	0.593	0.981	0.392

^1^ Standard error of the mean. ^2^ Firmicutes/Bacteroidetes Ratio.

## References

[1] Suchodolski J.S. (2011). Intestinal microbiota of dogs and cats: A bigger world than we thought. Vet. Clin. Small Anim. Pract..

[2] Mondo E., Marliani G., Accorsi P.A., Cocchi M., Di Leone A. (2019). Role of gut microbiota in dog and cat’s health and diseases. Open Vet. J..

[3] Pilla R., Suchodolski J.S. (2020). The role of the canine gut microbiome and metabolome in health and gastrointestinal disease. Front. Vet. Sci..

[4] AlShawaqfeh M.K., Wajid B., Minamoto Y., Markel M., Lidbury J.A., Steiner J.M., Serpedin E., Suchodolski J.S. (2017). A dysbiosis index to assess microbial changes in fecal samples of dogs with chronic inflammatory enteropathy. FEMS Microbiol. Ecol..

[5] Kim K.R., Kim S.M., Kim J.H. (2023). A pilot study of alterations of the gut microbiome in canine chronic kidney disease. Front. Vet. Sci..

[6] Thomsen M., Künstner A., Wohlers I., Olbrich M., Lenfers T., Osumi T., Shimazaki Y., Nishifuji K., Ibrahim S.M., Watson A. (2023). A comprehensive analysis of gut and skin microbiota in canine atopic dermatitis in Shiba Inu dogs. Microbiome.

[7] Ziese A.L., Suchodolski J.S. (2021). Impact of changes in gastrointestinal microbiota in canine and feline digestive diseases. Vet. Clin. N. Am. Small Anim. Pract..

[8] Gibson G.R., Hutkins R., Sanders M.E., Prescott S.L., Reimer R.A., Salminen S.J., Scott K., Stanton C., Swanson K.S., Cani P.D. (2017). Expert consensus document: The International Scientific Association for Probiotics and Prebiotics (ISAPP) consensus statement on the definition and scope of prebiotics. Nat. Rev. Gastroenterol. Hepatol..

[9] Markowiak P., Śliżewska K. (2018). The role of probiotics, prebiotics and synbiotics in animal nutrition. Gut Pathog..

[10] Said D.S., Chrismadha T., Mayasari N., Febrianti D., Suri A.R.M. (2022). Nutrition value and growth ability of aquatic weed *Wolffia globosa* as alternative feed sources for aquaculture system. IOP Conference Series: Earth and Environmental Science.

[11] Senayai A., Harnvanichvech Y., Vajrodaya S., Oyama T., Kraichak E. (2025). Genetic and morphological variation among populations of duckweed species in Thailand. Plants.

[12] Kawamata Y., Shibui Y., Takumi A., Seki T., Shimada T., Hashimoto M., Inoue N., Kobayashi H., Narita T. (2020). Genotoxicity and repeated-dose toxicity evaluation of dried *Wolffia globosa* Mankai. Toxicol. Rep..

[13] Boonarsa P., Bunyatratchata A., Chumroenphat T., Thammapat P., Chaikwang T., Siripan T., Li H., Siriamornpun S. (2024). Nutritional quality, functional properties, and biological characterization of watermeal (*Wolffia globosa*). Horticulturae.

[14] On-Nom N., Promdang P., Inthachat W., Kanoongon P., Sahasakul Y., Chupeerach C., Suttisansanee U., Temviriyanukul P. (2023). *Wolffia globosa*-based nutritious snack formulation with high protein and dietary fiber contents. Foods.

[15] Ruekaewma N., Piyatiratitivorakul S., Powtongsook S. (2015). Culture system for *Wolffia globosa* L. (Lemnaceae) for hygiene human food. Songklanakarin J. Sci. Technol..

[16] Montserrat-Malagarriga M., Castillejos L., Salas-Mani A., Torre C., Martín-Orúe S.M. (2024). Use of different synbiotic strategies to improve gut health in dogs. Animals.

[17] Jatuponwiphat T., Namrak T., Supataragul A., Nitisinprasert S., Nakphaichit M., Vongsangnak W. (2019). Comparative genome analysis reveals metabolic traits associated with probiotics properties in *Lactobacillus reuteri* KUB-AC5. Gene Rep..

[18] Sobanbua S., Tangthong J., Suveatwatanakul A., Nakphaichit M., Keawsompong S., Nitisinprasert S. (2020). Cloning and expression of the antimicrobial peptide from *Lactobacillus reuteri* KUB-AC5 and its characterization. Int. J. Agric. Technol..

[19] Mok K., Honwichit O., Funnuam T., Charoensiddhi S., Nitisinprasert S., Nielsen D.S., Nakphaichit M. (2024). Synergistic activity of *Limosilactobacillus reuteri* KUB-AC5 and water-based plants against *Salmonella* challenge in a human in vitro gut model. Sci. Rep..

[20] AOAC (2019). Official Methods of Analysis of the Association of Official Analytical Chemists: Official Methods of Analysis of AOAC International.

[21] Nitisinprasert S., Nilphai V., Bunyun P., Sukyai P., Doi K., Sonomoto K. (2000). Screening and identification of effective thermotolerant lactic acid bacteria producing antimicrobial activity against *Escherichia coli* and *Salmonella* sp. resistant to antibiotics. Agric. Nat. Resour..

[22] Nakphaichit M., Sobanbua S., Siemuang S., Vongsangnak W., Nakayama J., Nitisinprasert S.J.B.M. (2019). Protective effect of *Lactobacillus reuteri* KUB-AC5 against *Salmonella enteritidis* challenge in chickens. Benef. Microbes.

[23] Tantibhadrasapa A., Li S., Buddhasiri S., Sukjoi C., Mongkolkarvin P., Boonpan P., Wongpalee S.P., Paenkaew P., Sutheeworapong S., Nakphaichit M. (2024). Probiotic *Limosilactobacillus reuteri* KUB-AC5 decreases urothelial cell invasion and enhances macrophage killing of uropathogenic *Escherichia coli* in vitro study. Front. Cell. Infect. Microbiol..

[24] Pallin A. (2012). Lactobacilli in the Gastrointestinal Tract of Dog and Wolf. Master’s Thesis.

[25] Cline M.G., Burns K.M., Coe J.B., Downing R., Durzi T., Murphy M., Parker V. (2021). 2021 AAHA nutrition and weight management guidelines for dogs and cats. J. Am. Anim. Hosp. Assoc..

[26] Freeman L., Becvarova I., Cave N., MacKay C., Nguyen P., Rama B., Takashima G., Tiffin R., van Beukelen P., Yathiraj S. (2011). WSAVA nutritional assessment guidelines. J. Feline Med. Surg..

[27] De Coster W., Rademakers R. (2023). NanoPack2: Population-scale evaluation of long-read sequencing data. Bioinformatics.

[28] Curry K.D., Wang Q., Nute M.G., Tyshaieva A., Reeves E., Soriano S., Wu Q., Graeber E., Finzer P., Mendling W. (2022). Emu: Species-level microbial community profiling of full-length 16S rRNA Oxford Nanopore sequencing data. Nat. Methods.

[29] Sońta M., Rekiel A., Batorska M. (2019). Use of duckweed (*Lemna* L.) in sustainable livestock production and aquaculture–a review. Ann. Anim. Sci..

[30] Areerat S., Chundang P., Lekcharoensuk C., Patumcharoenpol P., Kovitvadhi A. (2023). Insect-based diets (house crickets and mulberry silkworm pupae): A comparison of their effects on canine gut microbiota. Vet. World.

[31] Dhamaratana S., Methacanon P., Tunsagool P., Nakphaichit M., Mok K., Honwichit O., Charoensiddhi S. (2025). Alterations in gut microbiome and metabolite profiling during in vitro fermentation of duckweed (*Wolffia globosa*) and its extracts by gut bacteria from obese adults. Future Foods.

[32] Meineri G., Saettone V., Radice E., Bruni N., Martello E., Bergero D. (2021). The synergistic effect of prebiotics, probiotics and antioxidants on dogs with chronic kidney disease. Ital. J. Anim. Sci..

[33] Kore K.B., Pattanaik A.K., Das A., Sharma K. (2012). Evaluation of mannanoligosaccharide as prebiotic functional food for dogs: Effect on nutrient digestibility, hind gut health and plasma metabolic profile. Indian J. Anim. Sci..

[34] Lin C.Y., Kerr K.R., Panasevich M.R., Daristotle L., Frantz N.Z. (2024). Duckweed protein as an alternative plant-based protein source for dog and cat dry diets. J. Anim. Sci..

[35] Qiao S., Liu C., Sun L., Wang T., Dai H., Wang K., Bao L., Li H., Wang W., Liu S.J. (2022). Gut Parabacteroides merdae protects against cardiovascular damage by enhancing branched-chain amino acid catabolism. Nat. Metab..

[36] Wang H., Feng L., Pei Z., Zhao J., Lu S., Lu W. (2025). Gut microbiota metabolism of branched-chain amino acids and their metabolites can improve the physiological function of aging mice. Aging Cell.

[37] Lee M.D., Pedroso A.A., Lumpkins B., Cho Y., Maurer J.J. (2023). Pioneer colonizers: Bacteria that alter the chicken intestinal morphology and development of the microbiota. Front. Physiol..

[38] Garcia-Mazcorro J.F., Dowd S.E., Poulsen J., Steiner J.M., Suchodolski J.S. (2012). Abundance and short-term temporal variability of fecal microbiota in healthy dogs. Microbiologyopen.

[39] Van Hul M., Le Roy T., Prifti E., Dao M.C., Paquot A., Zucker J.D., Delzenne N.M., Muccioli G.G., Clément K., Cani P.D. (2020). From correlation to causality: The case of Subdoligranulum. Gut Microbes.

[40] Wu Y., Liu Z., Zhang H., Zhou L., Liang W., Su P., Zeng Z., Zhao F., Qiu J., Chen C. (2026). Unraveling the Obesity-Combating Potential of *Parabacteroides goldsteinii* and *Bacteroides sartorii*: A Dual-Probiotic Approach. Probiot. Antimicrob. Proteins.

[41] Pinna C., Vecchiato C.G., Grandi M., Stefanelli C., Zannoni A., Biagi G. (2021). Seaweed supplementation failed to affect fecal microbiota and metabolome as well as fecal IgA and apparent nutrient digestibility in adult dogs. Animals.

[42] Rojas C.A., Park B., Scarsella E., Jospin G., Entrolezo Z., Jarett J.K., Martin A., Ganz H.H. (2024). Species-level characterization of the core microbiome in healthy dogs using full-length 16S rRNA gene sequencing. Front. Vet. Sci..

[43] Embree M., Gogul G., Yang F. (2024). Native Microbials Inc. Microbial Compositions and Methods of Use for Canine Enteropathy and Dysbiosis. U.S. Patent.

[44] Elsayed S., Zhang K. (2007). *Clostridium glycolicum* bacteremia in a bone marrow transplant patient. J. Clin. Microbiol..

[45] Jiang W., Abrar S., Romagnoli M., Carroll K.C. (2009). *Clostridium glycolicum* wound infections: Case reports and review of the literature. J. Clin. Microbiol..

[46] Van Leer C., Wensing A.M.J., Van Leeuwen J.P., Zandbergen E.G.J., Swanink C.M.A. (2009). *Clostridium glycolicum* isolated from a patient with otogenic brain abscesses. J. Clin. Microbiol..

[47] Segata N., Abubucker S., Goll J., Schubert A.M., Izard J., Cantarel B.L., Rodriguez-Mueller B., Waldron L., Zucker J., Thiagarajan M. (2011). Microbial community function and biomarker discovery in the human microbiome. Genome Biol..

[48] Longshaw M., Quest B., Miller W., Oba P.M., Swanson O.R., Swanson K.S., Miller K. (2025). The safety of FeedKind Pet^®^ (*Methylococcus capsulatus*, Bath) as a cultured protein source in the diet of adult dogs and its effect on feed digestibility, fecal microbiome, and health status. Animals.

[49] Pahalagedara A.S., Flint S., Palmer J., Brightwell G., Luo X., Li L., Gupta T.B. (2023). Non-targeted metabolomic profiling identifies metabolites with potential antimicrobial activity from an anaerobic bacterium closely related to *Terrisporobacter* species. Metabolites.

[50] Gagné J.W., Wakshlag J.J., Simpson K.W., Dowd S.E., Latchman S., Brown D.A., Brown K., Swanson K.S., Fahey G.C. (2013). Effects of a synbiotic on fecal quality, short-chain fatty acid concentrations, and the microbiome of healthy sled dogs. BMC Vet. Res..

[51] Tanprasertsuk J., Jha A.R., Shmalberg J., Jones R.B., Perry L.M., Maughan H., Honaker R.W. (2021). The microbiota of healthy dogs demonstrates individualized responses to synbiotic supplementation in a randomized controlled trial. Anim. Microbiome.

[52] Khan I., Bai Y., Zha L., Ullah N., Ullah H., Shah S.R.H., Sun H., Zhang C. (2021). Mechanism of the gut microbiota colonization resistance and enteric pathogen infection. Front. Cell. Infect. Microbiol..

