# Supplementary material for: Effects of Dietary Supplementation with Wolffia globosa and Limosilactobacillus reuteri KUB-AC5 on Health Parameters and Gut Microbiota Composition in Dogs
Source: Biology (Basel). 2026 Jul 3;15(13):1067. doi: 10.3390/biology15131067 (PMC13359790; doi:10.3390/biology15131067)
Supplement: Supplementary file 1 [file biology-15-01067-s001.zip › Supplementary File 1.pdf]

Supplementary File

Table S1. Animal Information and Signalment

Code	Dog's name	Group	Sex	Initial body weight (kg)	Initial body condition score (9-scale)
Dog01	Peper	CON	Female	11.5	3
Dog02	Chokedee	PRE	Male	20.2	5
Dog03	Dang lek	SYN	Male	15.5	3
Dog04	Nam choke	CON	Female	14.4	4
Dog05	Tungtao	PRE	Female	14.1	3
Dog06	Old joy	SYN	Female	14.3	5
Dog07	New joy	CON	Female	16	3
Dog08	Brown don	PRE	Female	11.8	3
Dog09	Khao	SYN	Female	16	6
Dog10	Song phoo	CON	Male	18.6	6
Dog11	Hao 1	PRE	Female	20	5
Dog12	Ta sarm	SYN	Male	15.8	3
Dog13	Lay khao	CON	Female	22.6	7
Dog14	Peepo	PRE	Male	19.5	4
Dog15	Pakmom	SYN	Female	18.3	4
Dog16	Black don	CON	Male	18.3	3
Dog17	Pui	PRE	Male	17.1	4
Dog18	Toob sai	SYN	Male	20.9	3
Dog19	Jao to	CON	Female	19.4	4
Dog20	Lhung	PRE	Male	11	3
Dog21	Sab	SYN	Female	16.6	5
Dog22	Jukkalan	CON	Female	14.2	3
Dog23	Brownie	PRE	Female	12.1	3
Dog24	Blackie	SYN	Male	19.7	3
