# Supplementary material for: Effects of Dietary Supplementation with Wolffia globosa and Limosilactobacillus reuteri KUB-AC5 on Health Parameters and Gut Microbiota Composition in Dogs
Source: Biology (Basel). 2026 Jul 3;15(13):1067. doi: 10.3390/biology15131067 (PMC13359790; doi:10.3390/biology15131067)
Supplement: Supplementary file 1 [file biology-15-01067-s001.zip › Supplementary File 2.pdf]

Supplementary File

Table S2. Sequencing depth statistics for all samples (Data Quality Control)

Code	Groups	Day	Raw Reads	Clean Reads	Mean_len(bp)	Mean_Q
D01CS	Control	0	100167	82960	1450	24.33
D02PS	Prebiotic	0	99628	83408	1447	24.29
D03SS	Synbiotic	0	99607	83426	1454	24.67
D04CS	Control	0	100130	84060	1443	24.79
D05PS	Prebiotic	0	99812	83635	1443	24.23
D06SS	Synbiotic	0	99847	82472	1452	24.33
D07CS	Control	0	100132	83330	1445	24.26
D08PS	Prebiotic	0	100074	81767	1453	24.39
D09SS	Synbiotic	0	100396	83106	1451	24.35
D10CS	Control	0	99754	89547	1447	25.53
D11CS	Control	0	99639	88817	1453	25.59
D12PS	Prebiotic	0	99694	88552	1444	25.42
D13SS	Synbiotic	0	99778	89074	1445	25.47
D14CS	Control	0	99443	88123	1445	25.96
D15PS	Prebiotic	0	96645	84236	1452	25.7
D16SS	Synbiotic	0	94467	83119	1443	25.51
D17CS	Control	0	96039	85337	1444	25.49
D18PS	Prebiotic	0	83249	71303	1446	25.55
D19SS	Synbiotic	0	77561	66382	1449	25.37
D20CS	Control	0	93226	82306	1442	25.83
D21PS	Prebiotic	0	93834	81903	1448	25.51
D22SS	Synbiotic	0	77322	67240	1447	25.54
D01CE	Control	28	82404	71853	1454	25.56
D02PE	Prebiotic	28	98734	86369	1446	25.51
D03SE	Synbiotic	28	99570	87758	1444	25.47
D04CE	Control	28	96251	84811	1454	25.61
D05PE	Prebiotic	28	85030	73424	1444	25.48
D06SE	Synbiotic	28	78799	68280	1448	25.78
D07CE	Control	28	88996	77782	1453	25.46
D08PE	Prebiotic	28	97451	86573	1444	25.52
D09SE	Synbiotic	28	97162	86457	1443	25.47
D10CE	Control	28	89412	78106	1446	25.43
D11CE	Control	28	99632	88442	1449	25.33
D12PE	Prebiotic	28	100364	88816	1445	25.53

Code	Groups	Day	Raw Reads	Clean Reads	Mean_len(bp)	Mean_Q
D13SE	Synbiotic	28	100012	89686	1444	25.42
D14CE	Control	28	96042	84529	1445	25.44
D15PE	Prebiotic	28	99489	87464	1452	25.75
D16SE	Synbiotic	28	100325	88775	1444	25.47
D17CE	Control	28	96253	82997	1444	25.48
D18PE	Prebiotic	28	99697	87912	1445	25.32
D19SE	Synbiotic	28	93827	82694	1446	25.55
D20CE	Control	28	88632	76364	1444	25.51
D21PE	Prebiotic	28	94820	83245	1463	25.69
D22SE	Synbiotic	28	100184	88594	1451	25.7
